# A Model Prison
*The New York State Reformatory in Elmira. By Alexander Winter, F.S.S. Original English Edition, published simultaneously with the German Edition, with a Preface by Havelock Ellis, author of "The Criminal." (London: Swan, Sonnenschein and Co.)


**Published:** 1892-04-02

**Authors:** 


### A Model Prison.*

"We have reformed our prisons; we have been content to look upon the prisoner as, literally, a cypher. He is merely an incident in a huge system of routine, to the efficient working of which he is subordinated." This sentence, taken from Mr. Havelock Ellis's preface to Mr. Alexander Winter's account of the Elmira Reformatory, tells the blot on our criminal administration, as the institution described in the volume tells of the boldest and most successful attempt yet made at an improvement in the method of dealing with that disagreeable and pitiable class. The New York State Reformatory at Elmira was founded in 1876 by Mr. G. S. Brockway, who is still its Director-General, its head, heart, and soul. The system carried out at Elmira, though elaborate in its details, is fundamentally very simple; it consists, in fact, in letting the prisoners work out their own liberation. The selection of inmates is limited to "males of sixteen to thirty years of age, who have fallen for the first time under the penal code," so that there is not the association of hardened sinners—criminals who have been so often convicted that the prison has become their most permanent home—to interfere with the efforts made by the directors to restore to the paths of rectitude and industry those who have fallen once. And all these efforts are founded on a thing as yet unknown in England—the indeterminate sentence.

The indeterminate sentence, already dealt with in these columns, does not wholly deserve its name. Literally, it should imply that every convicted criminal should be sentenced to imprisonment for life, subject to the possibility of regaining his liberty through his own good conduct. It is to be wished that this could indeed be done, for there are criminals who, though they are doggedly content to wait till the expiration of their two, five, or seven years' sentence bring the inevitable day of liberty—would yet tremble at the fearful indefiniteness of the sentence "for life." In practice, the indeterminate sentence means that the prisoner is sentenced for the maximum term allowed by the law for the offence for which he is convicted, with the chance of that period being shortened, according as he behaves during his detention.

Arrived at Elmira, the prisoner is allowed a few days of solitary reflection before he is brought into the presence of Mr. Brockway, who, by means of a searching, but skilful examination, informs himself of the past environment, hereditary instincts, and general condition—moral, physical, and intellectual—of the man committed to his care. In this examination lies the strength of Elmira, and, it is to be feared, the weakness of the system there so successfully carried on. For it depends on the character of one man—the general superintendent. Mr. Brockway is a man of keen insight as well as high ideals; his estimate of a prisoner's character may be taken as fundamentally correct; but if it were otherwise, an initial error might lead to fatal blundering in treatment. And men like Mr. Brockway are not to be found every day, nor picked up by advertising in the newspaper. A good deal certainly might be done by introducing a new notion of his duties into the head of the governor of every prison; but in the end the idiosyncrasy of the chief would undoubtedly affect the whole character of an institution, making it a living body or a dull machine.

There are at Elmira three grades of prisoners, rated according to their conduct. New comers are put in the second, or middle class, from which they may rise to the first or sink to the third grade. Deceit, even in trifles, quarrelling, especially if it leads to assault, and attempted flight invariably cause degradation to the third class, while negligence, careless work, laziness in the school or workshop—unless proved to be due to some physical defect for which the offender is not responsible—delay promotion to the first, and may eventually cause a fall to the third grade. The privileges of the first grade are so considerable, that any one whose sensibilities are not wholly dulled would naturally seek to attain to them. To receive and write letters every week, to keep their gas burning for half an hour after the other inmates, to be permitted free in tercourse with each other for an hour a day, and at all meal-times, to have these meals served on small tables with porcelain and white cloths, to be promoted to be guards and overseers, to have everything in dress and treatment that too harshly suggests the word "prisoner" kept out of sight, is a prospect that not only enlivens those who are not degraded, but awakens in them a sensitiveness to regularity and propriety of life which makes a return to the old haphazard existence of the criminal appear very undesirable. The diet list at first sight seems unduly luxurious. We can imagine the growling of the British taxpayer if he knew of "gaol-birds" having a *ménu* like this:—

"Breakfast: Beef hash, potatoes, bread, coffee, sugar."

"Dinner: Three times soup and meat, twice mutton stew, once beef and turnips and roast beef and gravy, always including bread. In addition, the first grade receives daily, and the second four times a week, coffee and sugar; and besides, the first grade sometimes receives dessert, preserved or dried fruit."

Mr. Brockway has—not unnaturally—been accused of extravagance in this matter. His reply was: "Good food is, for every one, of the first necessity for orderly life, if he wants to make the fullest possible use of the powers of his body, and my long experience has taught me that I obtain far better results with the subjects by supplying a good diet." After all there is something in it. No man can cherish the highest sentiments when he is hungry; and in criminals a poor, starved physique has often a subtle connection with the egotism, the discontent, and the covetousness that led them into sin. Physical culture of every kind occupies a prominent place in the economy of Elmira. Prisoners whose intellectual and moral consciousness it has been found difficult to arouse, are put through a course of gymnastics, and with the best possible results. Of all the industries which the prisoners are taught, farm labour is the most assiduously taught, so that physical health forms a sound basis for the instruction given in the school.

"School" does not, in Elmira, mean the three R's alone. Besides these, instruction is given in American history, English literature, physical geography, political economy, civil government, and practical ethics. Does this syllabus seem rather high flown? Before condemning it, one should remember that it is his unconscious acquaintance with the three last subjects that keeps the average man from crime. At any rate, the Elmira criminals appreciate the instruction, seem to understand and profit by it, and take a most energetic interest in the discussions on practical morality, which are held every week. The following letter from a prisoner shows that the Elmira criminal is not wholly destitute of a conscience:—

"Gen'l Sup't,—Please helow me to attend the lectures on practical morality Sundays fornoon. I ges I can pas examination. I would much like it, as I think morality is my weakest point."

To assist in making this weak point strong, the prisoners are taught some honest trade. It is unfortunately forbidden now to put prison labour into the open market—the law choosing to put the material interests of individual manufacturers before the moral well-being of the community at large—but an attempt is made to make Elmira self-supporting. It is not easy, for the prisoners do not, as a rule, remain long enough after they have learned a trade to repay the cost of instruction, but something has been done, and more is hoped for. One great advantage of the system of manual training is that the prisoner earns a wage, and when he regains his freedom, he begins life anew with a certain amount of useful knowledge, and a sum of money to keep him while he looks for work outside.

What is the result of this strange experiment—this treating of criminals like delicate children, this apparently

* The New York State Reformatory in Elmira. By Alexander Winter, F.S.S. Original English Edition, published simultaneously with the German Edition, with a Preface by Havelock Ellis, author of "The Criminal." (London: Swan, Sonnenschein and Co.)

expensive nurture of the ill grown plants in the forest of humanity? Of those who have left the institution some have been lost sight of. Of these, not all will have kept to the path of virtue; but we cannot assume that all have left it. And Mr. Brockway is able to show the clearest proofs of the satisfactory condition of over seventy-eight per cent. of his former subjects. More than three-fourths of a criminal community redeemed from evil! What other institution can show anything like this? If the publication of Mr. Winter's account of the Elmira Reformatory helps any such result on this side of the Atlantic, we shall give it a threefold welcome.

